# Machine learning analysis of microbial flow cytometry data from nanoparticles, antibiotics and carbon sources perturbed anaerobic microbiomes

**DOI:** 10.1186/s13036-018-0112-9

**Published:** 2018-09-12

**Authors:** Abhishek S. Dhoble, Pratik Lahiri, Kaustubh D. Bhalerao

**Affiliations:** Department of Agricultural and Biological Engineering, University of Illinois at Urbana-Champaign, 1304 W. Pennsylvania, Urbana, 61801 USA

**Keywords:** Flow cytometry, Machine learning, Microbial community fingerprinting, Pattern recognition, Anaerobic digestion, Deep learning

## Abstract

**Background:**

Flow cytometry, with its high throughput nature, combined with the ability to measure an increasing number of cell parameters at once can surpass the throughput of prevalent genomic and metagenomic approaches in the study of microbiomes. Novel computational approaches to analyze flow cytometry data will result in greater insights and actionability as compared to traditional tools used in the analysis of microbiomes. This paper is a demonstration of the fruitfulness of machine learning in analyzing microbial flow cytometry data generated in anaerobic microbiome perturbation experiments.

**Results:**

Autoencoders were found to be powerful in detecting anomalies in flow cytometry data from nanoparticles and carbon sources perturbed anaerobic microbiomes but was marginal in predicting perturbations due to antibiotics. A comparison between different algorithms based on predictive capabilities suggested that gradient boosting (GB) and deep learning, i.e. feed forward artificial neural network with three hidden layers (DL) were marginally better under tested conditions at predicting overall community structure while distributed random forests (DRF) worked better for predicting the most important putative microbial group(s) in the anaerobic digesters viz. methanogens, and it can be optimized with better parameter tuning. Predictive classification patterns with DL (feed forward artificial neural network with three hidden layers) were found to be comparable to previously demonstrated multivariate analysis. The potential applications of this approach have been demonstrated for monitoring the syntrophic resilience of the anaerobic microbiomes perturbed by synthetic nanoparticles as well as antibiotics.

**Conclusion:**

Machine learning can benefit the microbial flow cytometry research community by providing rapid screening and characterization tools to discover patterns in the dynamic response of microbiomes to several stimuli.

**Electronic supplementary material:**

The online version of this article (10.1186/s13036-018-0112-9) contains supplementary material, which is available to authorized users.

## Background

The science of microbial ecology is on the verge of revolution [1] partly due to the decreasing costs of next generation sequencing (NGS) [2] and the increasing popularity of flow cytometry microbial characterization [3, 4]. The current processing pipeline for NGS requires ~ 14 h compared to ~ 2 h for flow cytometry. At this time, flow cytometry is significantly more high throughput for resolving rapid dynamic changes in the structure and function of the microbial communities over time, which is particularly crucial for studying health and wellness in dynamic biosystems [5]. To keep up with the information throughput, there is a need to develop tools that measure microbiome features beyond genomic or functional diversity [6]. During the past 50 years, flow cytometry has been proven as an established tool for single cell analysis [7, 8]. Currently, there are over 100 companies in the flow cytometry business worldwide constituting more than $3 billion [9]. Since its genesis in 1965 [10] and increased popularity since the 1970s [11], the basic design of flow cytometers has remained almost unchanged, which emphasizes its technological robustness and is an ideal tool for building actionable solutions for microbiome research community.

Previously, we demonstrated the utility of flow cytometry in classifying microbial consortia based on morphological and metabolic characteristics complementing existing genomic technologies in rapid characterization of microbiome dynamics [12]. With advances in cytometry, the number of parameters that can be measured simultaneously from single particles has increased multifold; most notable of these advances are the addition of more powerful lasers [13]. Over the last decade, 18-parameter measurements [14] have given way to 30-parameter flow cytometers [15], with 100-parameter flow cytometry on the horizon [13]. High-throughput data acquisition, minimum sample preparation, and more parameters per cell, are now producing massive, high-dimensional datasets. Classical approaches to community ecology studies will need to be augmented with novel computational techniques to enable the analysis of these huge multidimensional datasets [16].

Similar to our previous demonstration [17], there are number of papers demonstrating the effectiveness of flow cytometry in the characterization of microbial community changes [18–22]. However, there are very few papers illustrating the possibility of the use of machine learning models to classify microbial samples studied by flow cytometry. A good overview of microbial flow cytometry fingerprinting literature, which deals with the extraction of variables based on flow cytometry data has been presented previously [23]. Similar methods have been proposed [24] and used [25] as data mining methods, from which resulting variables can be incorporated in machine learning models. Machine learning approaches have been attempted previously at the single-cell level for microbial flow cytometry data [26–28]. While most of these demonstrations were based on either staining microbial population of interest [29, 30] or attempting to analyze entire scatter pattern [26], it was evident from our prior demonstration [17] that with label-free flow cytometry parameters, it was possible to monitor and rapidly characterize the dynamics of complex anaerobic microbiomes associated with perturbations in its environmental factors.

Hence, here we demonstrate the use of open-source machine learning tools to analyze flow cytometry data generated in the anaerobic microbiome perturbation experiments exploiting the three-dimensional flow cytometry signals namely cell size (FSC or forward scatter), cell granularity/morphology (SSC or side scatter) and autofluorescence (corresponding to the same excitation/emission wavelength as in AmCyan standard dye). We also demonstrated for the first time, the use of unsupervised autoencoders for microbial flow cytometry data. Applications of the machine learning analysis of microbial flow cytometry data in potentially improving the performance of biodigesters, and to characterize the syntrophic resilience [31] of the microbial community structure when exposed to nanoparticles and antibiotics has also been demonstrated. Perhaps in the future such tools would enable futuristic endeavors such as waste treatment in long-term human spaceflight missions [32–34] or to transform human waste into food [35].

## Results

### Autoencoders as a powerful tool for anomaly detection in microbiomes perturbed with controlled carbon sources

**Fig. 1:**
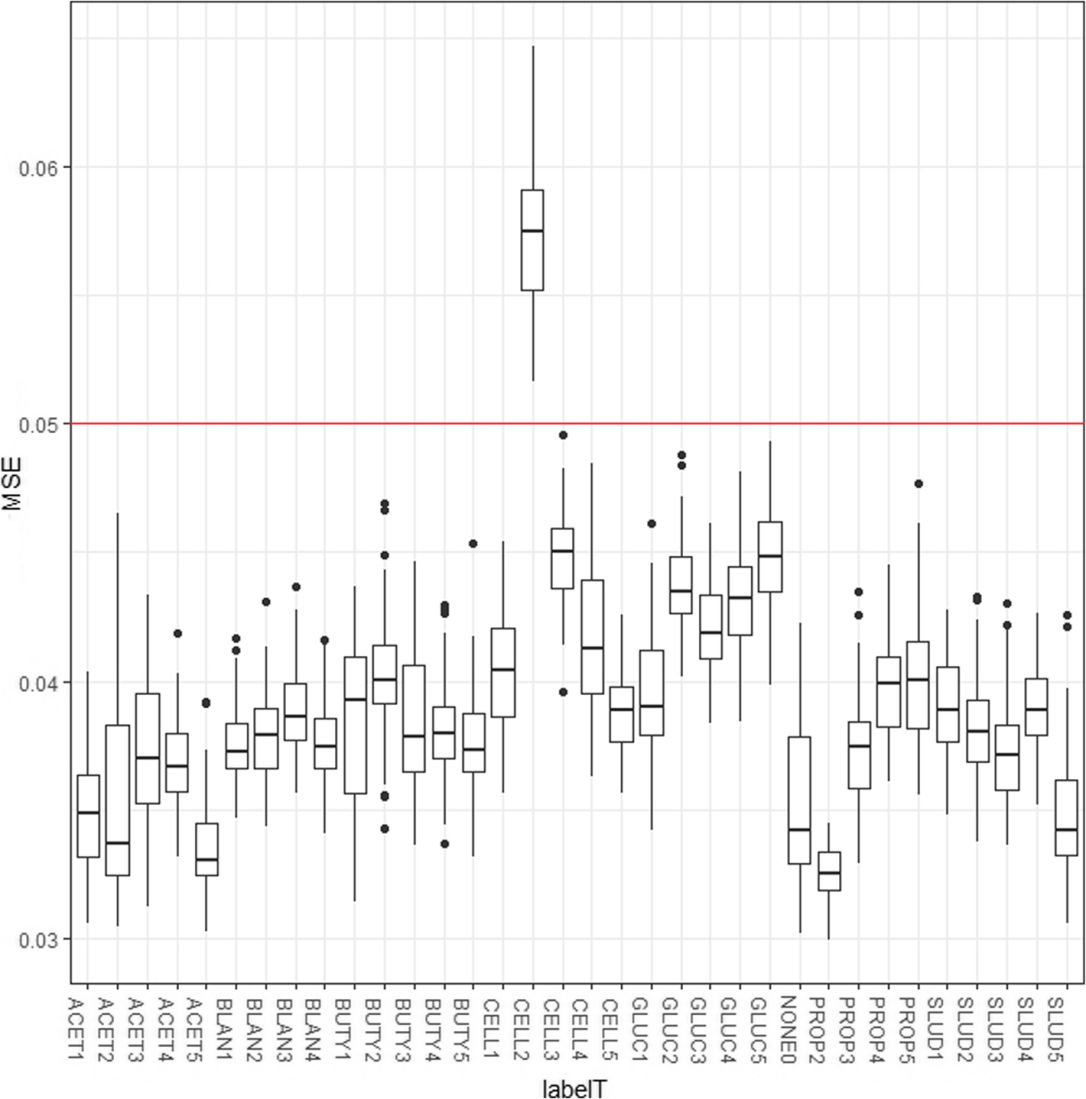
Unsupervised autoencoder analysis can be used to identify significantly perturbed microbiomes. The mean squared error (MSE) between actual value and reconstruction has been displayed on the y-axis for each sample tested. The red horizontal line at 0.05 MSE represents a threshold error to decide an outlier

Figure 1 shows the results from the h2o.ai’s unsupervised and non-linear autoencoder deep learning model. Communities perturbed with the controlled addition of GLUC and CELL looked different than the normal community and that with the controlled addition of other carbon sources. Interestingly, day 2 corresponded to peak biogas production for CELL at 272 mL while other days were in the range of 25–40 mL as shown in the Additional file 1: Figure S2. If this is compared with the clubbed predictions (Additional file 1: Figure S3), putative hydrolyzers (HYDRO) (i.e. CELL) were misclassified as putative acetogens (ACETO) which were same as CELL - PROP clubbing observed previously [17] and in the biogas plots (Additional file 1: Figure S2). Putative acidogens (ACIDO) (i.e. GLUC) and ACETO (i.e. PROP and BUTY) were also predicted well. These syntrophic acetogenic communities are believed to be very important in maintaining stable and robust anaerobic operation [36, 37].

### Putative functional group-level comparison with different machine learning models

**Fig. 2:**
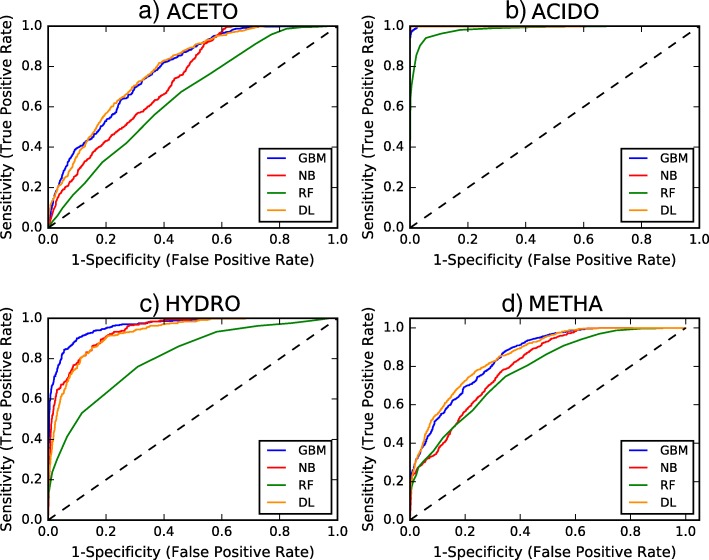
Receiver Operating Characteristic (ROC) curves comparing Gradient Boosting (GB), Naïve Bayes (NB), Distributed Random Forests (DRF) and Deep Learning (DL) (feed forward artificial neural network with three hidden layers) models on classification of (**a**) Acetogens (ACETO) (**b**) Acidogens (ACIDO) (**c**) Hydrolyzers (HYDRO) (**d**) Methanogens (METHA)

Table 1 lists prediction accuracies for the different models in the h2o.ai’s supervised learning data science algorithms suite. All models were evaluated on the test set (25% of the dataset). The performance of the models has been reported under tested conditions as described in ‘Methods’ section (hyperparameter values in Additional file 1: Section A2), and it can be optimized with better parameter tuning. Gradient boosting (GB) performed slightly better overall than deep learning (DL) (feed forward artificial neural network with three hidden layers) with 71.26% and 70.55% prediction accuracies respectively under tested conditions. Both Naïve Bayes (NB) and distributed random forests (DRF) had lower overall prediction accuracies on test sets (60.44% and 53.55% respectively) under tested conditions. From the area under the curve (AUC) plots shown in Fig. 2 and reported values in Table 2, DL (feed forward artificial neural network with three hidden layers) consistently outperformed all the other methods evaluated under tested conditions and marginally better than GB for the most important classes in anaerobic bioreactors (ACETO and METHA) [38–40], indicating that a single feed forward artificial neural network with three hidden layers model could be tuned for this task. The results were in concordance with similar supervised model comparison studies where feedforward neural nets have been concluded to perform best among various algorithms [41, 42].

**Table 1:** Machine learning model comparison (values in the boxes are prediction accuracies on test data; higher values are better) (^*^ Demonstrated deep learning model was a feed forward artificial neural network with three hidden layers)

Putative Groups	Gradient Boosting	Naïve Bayes	Distributed Random Forests	Deep Learning^*^
Acetogens	41.87%	63.87%	18.00%	52.67%
Acidogens	91.20%	97.07%	53.07%	99.73%
Hydrolyzers	65.60%	67.20%	10.67%	57.07%
Methanogens	85.17%	44.75%	89.33%	76.83%
Overall	71.26%	60.44%	53.55%	70.55%

**Table 2:** Area under the curve (AUC) values corresponding to Receiver Operating Characteristics (ROC) curves shown in Fig. 2 for test data (^*^ Demonstrated deep learning model was a feed forward artificial neural network with three hidden layers)

Putative Groups	Gradient Boosting	Naïve Bayes	Distributed Random Forests	Deep Learning^*^
Acetogens	0.7829	0.7279	0.6482	0.7853
Acidogens	0.9993	0.9999	0.9833	0.9983
Hydrolyzers	0.9638	0.9391	0.8055	0.9269
Methanogens	0.8520	0.8024	0.7773	0.8585

The limitations of the DL (feed forward artificial neural network with three hidden layers) in predicting methanogens (Table 1) were also evident in the clubbed prediction as shown in the Additional file 1: Figure S2 where putative methanogens (METHA) contradicted the expectations. Additionally, to ensure that we are not overfitting our models, we also trained them with 5-fold cross validation (results in Additional file 1: Table S4). The accuracy scores from this exercise followed similar trends, indicating that our models were rather stable and not overfitted.

### Predictive capabilities with deep learning (feed forward artificial neural network with three hidden layers)

**Fig. 3:**
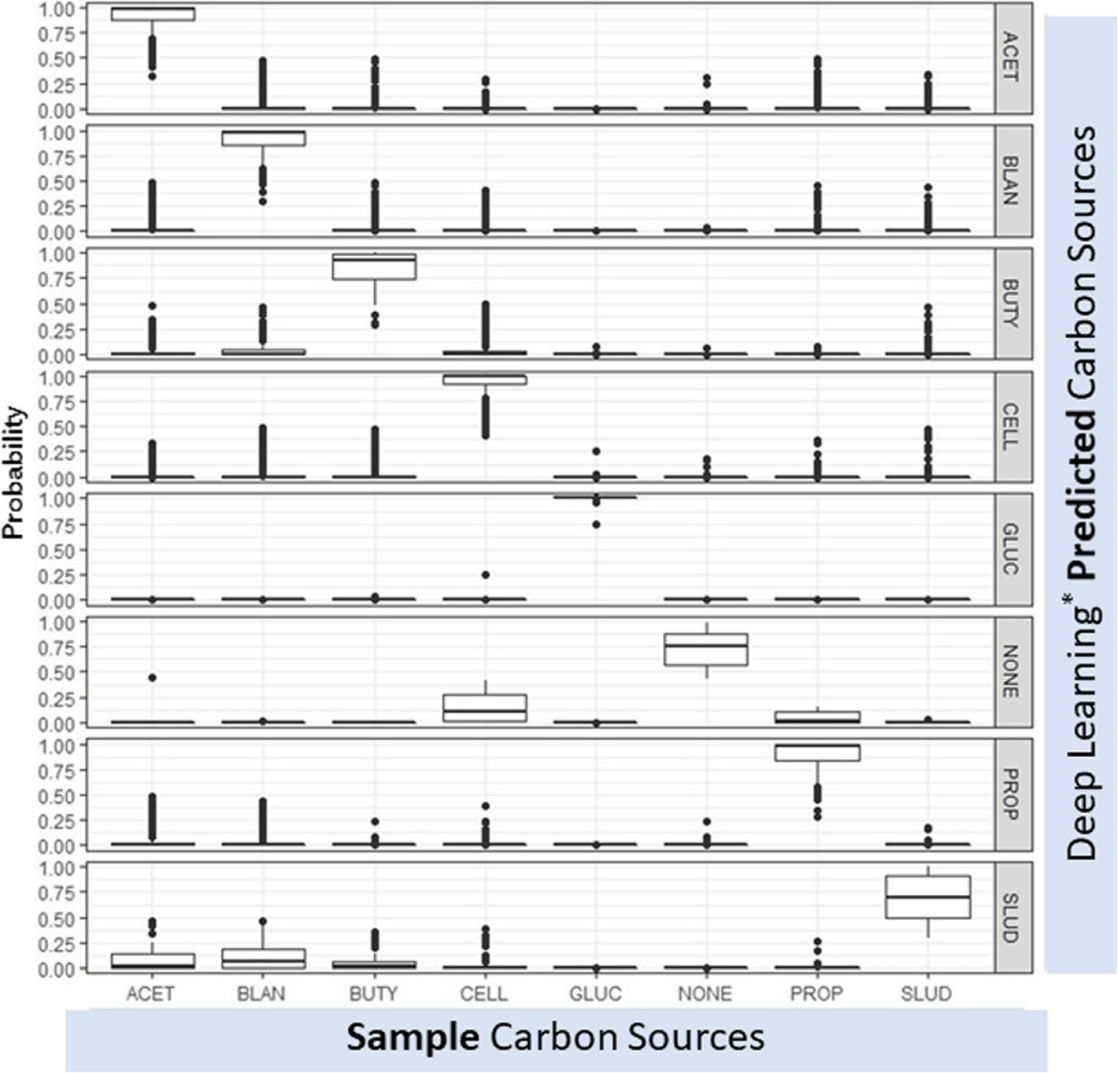
Box plots of the deep learning (^*^feed forward artificial neural network with three hidden layers) classification probabilities for carbon source

Since DL is increasingly popular because of its facility in handling large amount of data [43], and since one of the objectives of this paper is to illustrate the possibility of classifying large flow cytometry data sets in the field of anaerobic microbiology using machine learning for routine on-site microbiomes analysis, we examined its performance in classifying ‘sample vs predicted’ carbon sources. Figure 3 shows the results of the supervised DL model (feed forward artificial neural network with three hidden layers). In accordance with the results from classical multivariate analysis listed in our previous publication which showed that glucose (GLUC) looked very different from the rest [17], the classification probability for GLUC was best amongst tested carbon sources, followed by propionate (PROP) followed by sludge (SLUD). The variable importance output from h20.ai’s Flow platform reported in Additional file 1: Figure S7 shows that forward scatter (FSC) variables (<V1000) stood out to be the most relevant in DL model (feed forward artificial neural network with three hidden layers). As demonstrated in the multivariate analysis on microbial flow cytometry data in our previous publication [17], GLUC showed a distinct pattern in reaching a stable extremum in the colony structure, which was a point corresponding to minimum change. Additionally, both the peak and non-peak GLUC samples clustered separately in the multidimensional spacing (MDS) plot published previously [17], which was in accordance with the results presented here from the DL model (feed forward artificial neural network with three hidden layers). Further, blank samples (NONE), acetate (ACET) and sludge (SLUD) clustered separately in flow cytometry MDS plot [17] which resembled to NONE, ACET and SLUD misclassification evident in Fig. 3.

In the additional experimental set, when the cytometric fingerprints from individual carbon sources namely glucose (GLUC), cellulose (CELL), propionate (PROP), butyrate (BUTY), acetate (ACET) were trained with cytometric fingerprints from all carbon sources combined (WFED) and that from the starved community (STVD) samples. The expectation was that well-fed (WFED) community would display some degree of misclassification towards one of the five carbon sources. However, to our surprise, WFED community looked totally different than the rest and was more like STVD than individual carbon sources (confusion matrix in Additional file 1: Table S3, associated plot in Additional file 1: Figure S10). Furthermore, to evaluate the specificity of the models, a completely new carbon source in the form of newsprint waste (NEWS) was evaluated. Data from NEWS samples were trained with the other individual carbon sources data. Considering the primary composition of newspaper waste to be cellulosic fibers, the expectation was that it would display some similarity with CELL. Once again to our surprise, NEWS community looked totally different than rest of the carbon sources. These results point towards the uniqueness of flow cytometry signatures from complex carbon sources perturbed microbiomes in anaerobic digesters.

### Applications in elucidating functionally redundant microbiomes

To test the practical utility of the best performing DL model (feed forward artificial neural network with three hidden layers) beyond carbon sources, model implementation exercises were carried out with a separate set of perturbations namely a) nanoparticles and b) antibiotics. Interestingly even though the community looked quite different from one another, none showed any toxic impact on anaerobic digestion process observed in terms of biogas production, methane composition, Total Chemical Oxygen Demand (TCOD)/ Soluble Chemical Oxygen Demand (SCOD) reduction and Volatile Fatty Acids (VFA) accumulation (data not presented due to non-significance) with respect to both positive and negative controls for the nanoparticles and antibiotic (tetracycline) tested.

**Fig. 4:**
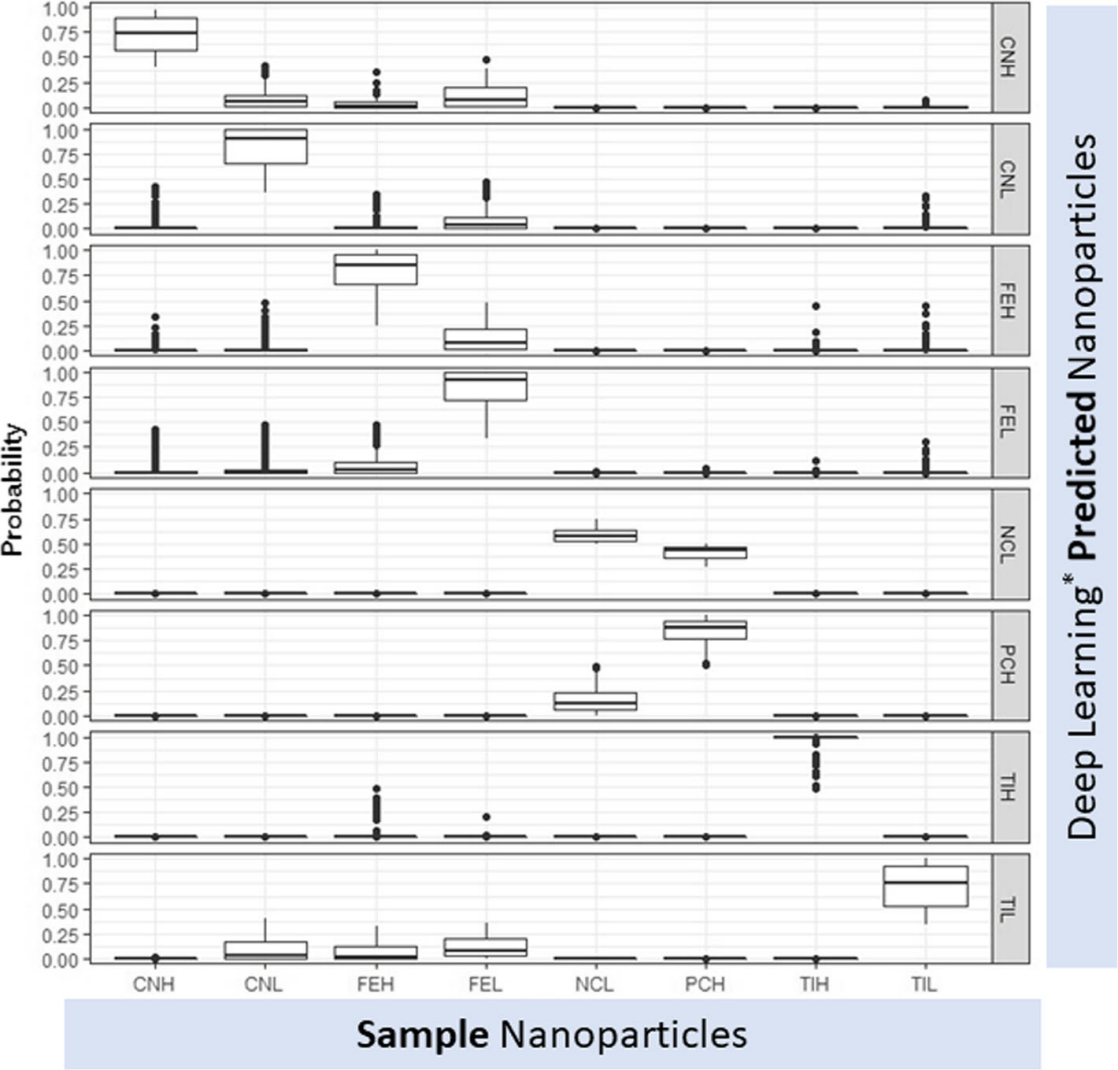
Box plots of deep learning (^*^feed forward artificial neural network with three hidden layers) prediction probabilities for nanoparticle-perturbed communities

The deep learning (feed forward artificial neural network with three hidden layers) analysis of flow cytometry data of nanoparticles perturbed community is shown in the Fig. 4. As evident, each nanoparticle-perturbed community looked different from one another and hence got predicted very well. TIH and TIL which were titanium (IV) oxide nanoparticles (TINPs) in high and low concentrations respectively have shown a little deviation and got misclassified as ferrous nanoparticles (FENPs) community in a few instances. The positive control (PCH) and negative control (NCH) got classified with less prediction scores when trained with nanoparticles than carbon sources. The community shifts in the context of community morphology were severe under nanoparticles compared to carbon sources.

**Fig. 5:**
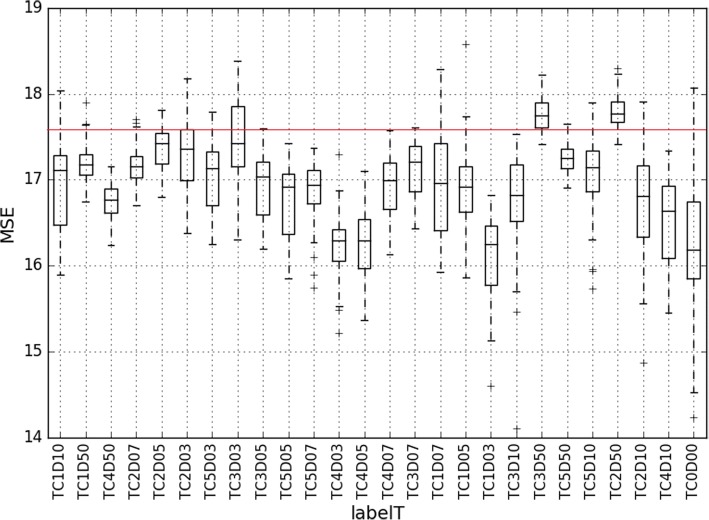
Unsupervised autoencoder analysis on antibiotics perturbed communities. The mean squared error (MSE) between actual value and reconstruction has been displayed on the y-axis for each sample tested. The red horizontal line at 17.5 MSE represents a threshold error to decide an outlier

Unsupervised autoencoders were also found to be marginally useful in predicting structural changes in the antibiotic-perturbed community. As shown in Fig. 5, literature [44] reported toxic level for tetracycline (TC2) and higher dosage (TC3) on day 50 got misclassified than the rest. Furthermore, TC2 might have started displaying maximum perturbation effects right from d 5 as evident from the misclassification instance for TC2D05 in Fig. 5. Both biogas (Additional file 1: Figure S8) and MDS (Additional file 1: Figure S9) plot on tetracycline perturbed samples displayed differences in community from day 0 and day 50. In the unsupervised autoencoder analysis, the community perturbed with higher concentration of TINPs on both the time points and higher concentration of carbon nanotubes (CNTs) on earlier time point looks different than the normal community (Additional file 1: Figure S4).

## Discussion

The machine learning approaches to the microbial flow cytometry dataset are still in infancy but the results presented here from the analysis of 1,500,000 microbes corresponding to each perturbation incidence (nanoparticles, antibiotics and carbon sources) demonstrate that it has much greater potential compared to the tradition microbial ecology statistical analysis like MDS as demonstrated in our previous publication [17] or similar Principal Component Analysis (PCA). Furthermore, our demonstration with five-fold cross validation assures the stability of various machine learning models against potential biases. With the advancement in the high-throughput nature of flow cytometry, combined with the increasing capacity to measure more cell parameters at once, massive and high-dimensional datasets on a routine daily basis would be generated in future and would add to the predictive power of the machine learning based approach [28].

Even though dimensionality reduction was not an objective, autoencoders were found to be powerful in learning a lower dimensional representation of the data. We do not suggest it replace dimensionality reduction analysis, nor do we intend it as a preprocessing step for DL models. Our work using autoencoders suggests that tools like the autoencoders can identify perturbations and hence may be valuable in stochastic optimization of the classical process design in the field of bioprocess engineering. While the current paradigm of process design is based on throughput rate, process yield and product purity [45], with the novel combination of cytometric fingerprinting and machine learning like the demonstrated autoencoders, process engineers may be able to optimize the digesters in real time, bypassing conventional lab-pilot-operations route [46].

The motivation behind the model comparison exercise was to see if one algorithm was better at predicting putative microbial group phenotypes than other. The efficiency in terms of speed and resources for model optimization, training and validation would be altogether different track of inquiry and it was not used for comparison in the proposed approach. AUC measured the probability that given two random points, one that is positive and other negative for the class of interest, the classifier scored the positive point higher than the negative one [47]. Considering the size and complexity of microbial flow cytometry data in anaerobic perturbations experiments, AUC acted as a yardstick for comparing the performance of the machine learning models used in the present study. Discrepancies in the trends between Tables 1 and 2 can be attributed to the fact that accuracies were computed at the set threshold values, while AUCs were computed by adding all the accuracies computed for all the possible threshold values. The ROCs in Fig. 2 could be perceived as an average (expected value) of those accuracies computed for all threshold values.

Under tested conditions, DRF worked better for putative methanogens, however the performance can be optimized with better parameter tuning. DRF generated a forest of classification (or regression) trees, rather than a single classification (or regression) tree. Each of these trees was a weak learner built on a subset of rows and columns [48]. More trees would have reduced the variance [49]. Both classification and regression took the average prediction over all their trees to make a final prediction, whether predicting for a class or numeric value [49]. Physiologically it may be too short of a time to get distinguishing phenotypic signatures for DL algorithm at the single cell level for methanogens [21, 50]. The stability of bioreactors is dependent on controlling acid build up and acid removal [51]. With slightly better AUC values for METHA and ACETO, DL seemed to satisfactorily perform under tested conditions in detecting perturbations in anaerobic bio-digesters. As demonstrated in Additional file 1: Figure S7, FSC variables (depicting size and morphology of individual cells) were the most important variables and DL appeared to perform better under tested conditions at classifying cells based on its size and morphological feature [19]. The tunability of a single DL model for multiple putative groups makes it computationally convenient for routine on-site microbiomes analysis.

The substrate composition determines the microbial community composition and organization [52, 53]. Indeed, it not only defines reactor conditions [54], but also provides the introduction of new species that are present in the substrate matrix, as, for instance, is the case for manure and waste activated sludge [55]. We were surprised that the samples fed with all five carbon sources at once did not get classified as one of the carbon sources. There might be a physiological explanation for different than normal behavior as reported by previous studies focused on studying structure-function relationships of anaerobic microbiomes [56–59]. *Smithllela*, *Syntrophobacte*r, and *Pelotomaculum* might have the ability to break down CELL faster than classical hydrolyzers like *Clostridium* or have greater doubling time [60]. PROP species predominantly fall in the genera *Smithllela*, *Syntrophobacter*, and *Pelotomaculum* [61, 62] while BUTY species in the genera *Syntrophus* and *Syntrophomonas* [63]. There have been reported phenotypic/physiological similarities between species of these two genera that may explain this trend [64, 65].

The composition of newspaper is reported as cellulose (glucose polymer), wood fiber (with 65.8% glucose, 19.8% xylose, 12.5% galactose and 1.3% mannose) [66]. The newspaper waste (NEWS) was a different type of carbon source compared to the earlier experiments with lab grade carbon sources. We were surprised that NEWS did not get misclassified as either CELL or GLUC but was distinctly predictable. It is possible that distinct groups of hydrolyzers and acidogens might be involved in initial degradation of newsprints than those feeding on pure cellulose or glucose [66, 67]. The accurate classification of various group of putative hydrolyzers and acidogens might become valuable in the routine monitoring of the anaerobic digesters in near future [68].

Even though the present results and the current associated literature [69] suggests no quantifiable toxicity of some nanoparticles on anaerobic digestion, the effect of NP-solvents was sometimes more significant than that of the NPs themselves - a point that may be of special interest for future nanotoxicological studies. The absence of observable toxicity from the exposure to tetracycline in terms of physico-chemical performance of the anaerobic culture was surprising. Both the nanotoxicity and antibiotics perturbation experiments were designed considering the current environmentally relevant concentrations [69]. With nanoparticles finding wider application in industrial products, such as antibacterial coatings, catalysts, biomedicine, skin creams and toothpastes, the magnitude of environmentally relevant concentrations may change in the future. Similarly, antibiotics can create perturbations and change the dynamics of the complex anaerobic microbial community. The present exercise demonstrates that flow cytometry can be used to monitor shifts away from normal microbial patterns. Our results suggest that even though the physiochemical parameters are not detectably different, changes in the community structure may be indicative of a community that may eventually break down.

## Conclusion

Autoencoders were found to be powerful in detecting anomalies in flow cytometry data from nanoparticle- and carbon source-perturbed anaerobic microbiomes but marginally so for antibiotic-perturbed communities. Anaerobic microbiomes displayed functional redundancy under nanotoxicity and antibiotic perturbations. Predictive classification patterns with supervised feed forward artificial neural network with three hidden layers were found to be robust. Model comparison exercise based on predictive capabilities concluded that under tested conditions, gradient boosting (GB) and deep learning (DL) (feed forward artificial neural network with three hidden layers) were marginally better at predicting overall community structure while distributed random forests (DRF) worked better at predicting methanogens, and it can be optimized with better parameter tuning. Flow cytometry data generated in various anaerobic microbiome perturbation experiments could be analyzed by various machine learning approaches for actionable insights.

## Methods

### Experimental set-up

The source of anaerobic culture, controlled perturbations and flow cytometric analysis were same as described in previously [17]. In summary, each of 100 ml of the triplicate mesophilic anaerobic microbiome batch assays were perturbed with different carbon sources separately (part of the dataset published previously [17]) and combined (new experiments). In addition, separate controlled perturbations were carried out in each of 100 ml of the triplicate mesophilic anaerobic microbiome batch assays with different nanoparticles, tetracycline antibiotics and newsprint waste.

#### Carbon sources

Individual carbon sources with 2000 mg/L chemical oxygen demand (COD) of glucose (GLUC), cellulose (CELL), propionate (PROP), butyrate (BUTY), acetate (ACET) separately as well as five of them combined (WFED) were added to individual batch assays. The chemical properties of the sludge (SLUD) used in this research are summarized in Additional file 1: Table S1. The peak day samples corresponded to the maximum biogas production observed (Additional file 1: Figure S2). Samples with no additional carbon sources were represented as NONE or starved (STVD). The experimental design has been shown in the Additional file 1: Figure S1. The data with individual carbon sources has been published previously [17]. The data with the combined five carbon sources as well as nanoparticles, tetracycline antibiotics and newsprint waste were from the separate unpublished experiments.

#### Conceptual division of putative microbial group(s)

For the clubbed flow frame analysis, the sample names were clubbed as per the conceptual division of putative microbial groups in anaerobic digestion process. The four groups were: (1) Putative hydrolyzes represented by “HYDRO” which were samples fed with cellulose (CELL) (2) Putative acidogens represented by “ACIDO” which were samples fed with glucose (GLUC) (3) Putative syntrophic acetogens represented by “ACETO” which were clubbed samples fed with propionate (PROP) and butyrate (BUTY) individually (4) Putative methanogens represented by “METHA” which were clubbed samples fed with acetate (ACET), sludge (SLUD) individually as well as those fed with no carbon source (BLAN and NONE). The reason behind clubbing ACET, SLUD, BLAN and NONE into METHA putative group was because all these samples were supposedly methanogenic samples with acetate utilizing methanogens were the predominant group in the waste water treatment plant anaerobic digesters [70].

#### Newsprint wastes

The feedstock used for the lab tests for the proposed campus anaerobic digester was newsprint from day-old *Daily Illini* Newspaper obtained from the waste sorting facility. Newsprint samples were either ground to powder, shredded to strips in an office paper shredder or whole pieces hand cut to size for required weight in each experiment. The goal was to predict the newspaper cytometric fingerprints (abbreviated as NEWS) off the carbon sources dataset generated with the proposed flow cytometry methodology.

#### Nanoparticles

For the nanotoxicity experiments, the sludge samples (SLUD) were mixed with various nanoparticles in low (L) 1 mg/g-TS (total solids) and high (H) 10 mg/g-TS concentrations. Plain sludge with anaerobic inoculum from active anaerobic digesters but no nanoparticles added was set up as a positive control (PCH) and anaerobic inoculum from the active anaerobic digesters without sludge or nanoparticles was set up as a negative control (NCL). The alleged toxic impact of nanoparticles on anaerobic digestion process was quantified by monitoring the biogas production daily along with gas composition, TCOD/ SCOD reduction and VFA production on biweekly basis. Drawing the samples for flow cytometric analysis every week helped build the cytometric fingerprints of the perturbed community, which forms the basis for subsequent machine learning analysis. TINPs are titanium (IV) oxide, anatase nanopowder, < 25 nm particle size, 99.7% trace metals basis, specific surface area 45–55 m^2^/g obtained from Sigma Aldrich (Catalog # 1317-70-0). FENPs are iron nanopowder, 25 nm average particle size, 99.5% trace metals basis from Sigma Aldrich (Catalog # 7439-89-6). CNTs are multi-walled carbon nanotube which are reported thin and short, < 5% metal oxide powder also from Sigma Aldrich (Catalog # 308068–56-6).

#### Antibiotics

For the antibiotics perturbation experiments, the sludge samples (SLUD) were mixed with tetracycline (Empirical Formula - Hill Notation: C22H24N2O8·xH2O, Molecular Weight: 444.43 anhydrous basis) obtained from Sigma Aldrich (CAS Number: 60–54-8). Tetracycline was reported to be profound inhibitors of anaerobic digestion, inhibiting methane production by up to 50% with a concentration of 40 mg/L [44]. Hence, to generate varying datasets for machine learning analysis of microbial samples, tetracycline with 20 mg/L (TC1), 40 mg/L (TC2) and 80 mg/L (TC3) concentrations were used along with positive (TC4) and negative (TC5) controls as described in the previous section.

### Flow cytometry analysis

Samples of the anaerobic microbial communities were collected from each serum bottle via a syringe with a 18 gauge needle (every 24 h following a biogas measurement for carbon sources). Initial sample (NONE_0) was the same for all the assays, which was the fresh inoculum. 750 μL of sample from each serum bottle was strained prior to flow cytometry using BD Falcon 12 × 75 mm Tube with Cell Strainer Cap having a 35 μm nylon mesh (Catalog No. 352235). The strained samples were suspended in Phosphate-buffered saline (PBS)-1X. Analyses were performed immediately on a Bio-sciences LSR II Flow Cytometry Analyzer. The excitation laser was tuned for 405-nm. Autofluorescence was measured as light passing a 450/50 photomultiplying tubes (PMT) and band pass filter with no long pass dichroic mirror. Signals were amplified with a 4-decade log amplifier and collected at a rate of approximately 1000 events per second. The fixed gating template as shown in the Additional file 1: Figure S6 (a) was used based on the control beads run (Additional file 1: Figure S6 (b)). Background events corresponding to dead/junk cells along with high FSC events (putative doublets) were excluded from the analysis (Additional file 1: Figure S6 (c)). Total 100,000 events collected for each sample were stored in corresponding Flow Cytometry Standard (fcs) files.

### Training and test dataset creation

For the machine learning based analysis, the fcs files obtained from a BD Bioscience LSR II Flow Cytometry Analyzer were imported. Each file comprised of 100,000 events representing 100,000 cells. The fcs file contained values for multiple parameters for each cell. But for our analysis we only chose “FSC-A”, “SSC-A” and “AmCyan-A” because in our previous publication [17], FSC or forward scatter, SSC or side scatter representing cell size, granularity/morphology respectively and autofluorescence (corresponding to the same excitation/emission wavelength as in AmCyan standard dye) were found to have maximum information. So, from each fcs file we get a matrix of 100,000 events with 3 columns (“FSC-A”, “SSC-A” and “AmCyan-A”) each.

Classifying single cells (also known as ‘events’) was out of purview of present study. Rather, we were interested in classifying distributions of events to understand microbial community dynamics specific to each perturbation. Our data was normally distributed in the measured flow cytometry values, hence we decided to combine 1000 events into a data point or a vector of 3000 values (1000 events comprising of 3 values corresponding to “FSC-A”, “SSC-A” and “AmCyan-A”) because the Wald Type confidence interval [71] with 3% error required a sample size of 1000. Additionally, the distribution of “FSC-A”, “SSC-A” and “AmCyan-A” values for all methanogens groups in the test set (described below) as well as the distribution of the same values in one randomly chosen vector of 1000 events has been presented in Additional file 1: Section A.3. Upon visual inspection, it is evident that the vector of 1000 events was able to capture the complexity and discriminative information of single cell variability in the population. There have been various workflows proposed for the data processing, feature extraction and data analysis of cytometric fingerprints applying Dalmatian Plot, CHIC, CyBar, or FlowFP [23], however majority of these were tested on the 2-dimenstional cytometric fingerprints, staining with one or more dyes. Previously, we have demonstrated correlation between prevalent community fingerprinting techniques like automated ribosomal intergenic spacer analysis (ARISA) and label-free, raw 3-dimensional cytometric fingerprints based on “FSC-A”, “SSC-A” and “AmCyan-A” [17]. The focus of this paper is to demonstrate the fruitfulness of machine learning algorithms in analyzing such label-free, raw 3-dimensional cytometric fingerprints. The presented framework could also potentially be applied to other approaches like Dalmatian Plot, CHIC, CyBar, or FlowFP from the flow cytometry fingerprinting literature to extract distribution-level features and/or with distribution level statistics such as the mean, standard deviation, percentile values etc.

Since there were 100,000 events in each fcs file, we got 100 such vectors. As described, we conducted each experiments in triplicate ranged over five time points, we had 1,500,000 (300,000 × 5) events for each perturbation incidence (carbon sources, nanoparticles, antibiotics) thereby generating a rich dataset with 1500 vectors representing each label. This dataset was then split into 75% training (1125 vectors) and 25% test (375 vectors) sets for each label. We decided to create a larger training split compared to testing since it is customary practice in machine learning community [72]. Additionally, we performed grid search (hyperparameters are reported in Additional file 1: Section A2) with nested cross-validation (four-folds outer and three-folds inner) on the training set (75% of dataset) to tune the hyperparameters [73]. The outer-fold creates non-overlapping datasets to evaluate the results of the inner-fold grid search cross validation. The best hyperparameters were selected based on the accuracy scores and standard deviation on the non-overlapping datasets in the outer-fold. The best hyperparameters from nested cross-validation were evaluated on the test set (25% of dataset).

An h2o.ai server was set-up as per instructions [74] (http://www.h2o.ai/) and the data was uploaded in individual comma-separated values (CSV) files. The fcs files and associated code are available at: https://github.com/adhoble/CFML-Perturb. The fcs files were also submitted to the Flow Repository (https://flowrepository.org/). (Repository ID: FR-FCM-ZYK4).

### Explanation of machine learning models

#### Autoencoders

An autoencoder learns a lower dimensional representation of the data by trying to learn an approximation of the identity function [75]. It is a feed-forward neural network where the hidden layer(s) compress(es) the input data and the output layer attempts to reconstitute the input data from the compressed encoding. This compressed encoding is the representation of the input data. This method can be used for anomaly detection such as email spams or financial frauds using the reconstruction mean squared error (MSE) on new data [75]. The MSE is defined by L (x, x’) = || x -x’||^2^ where x is the input vector (true value) and x’ is the vector reconstructed by the autoencoder (estimated/predicted value). Higher the MSE value, more anomalous a sample in relation to the pattern found in a whole dataset and the threshold MSE would vary with the particular perturbation experiment under consideration.

In our implementation of autoencoders, the h2o.ai function ‘deeplearning’ was used with ‘autoencoder’ option turned on and one hidden layer of 2000 nodes.

#### Deep learning (feed forward artificial neural network with three hidden layers) (DL)

A feed forward artificial neural network model or a deep neural network model is an arrangement of layers of neurons (inspired by biological neurons in human brain) with weighted connections such that there are no loops [76]. These neurons have activation thresholds which, if exceeded by a linear combination of the weight associated with incoming connections and data passed to them, are fired. During training, this model learns the weights of the connections to approximate any general target function.

In our implementation of deep learning (feed forward artificial neural network with three hidden layers), the default rectifier with dropout was selected for activation. The network contained input dropout ratio of 0.1, hidden dropout ratios of 0.2, 0.2, 0.1 and a default hidden layer sizes 2000,1000,500 were used. Number of epochs (i.e. number of times to iterate the dataset) were selected to 10. To add stability and improve generalization, either L1 or L2 regularization was used with the value of 1e-5 each.

#### Distributed random forests (DRF)

Random forests are type of ensemble machine learning methods commonly used for classification and regression. They extend decision trees by training many trees on random subsets of training data sampled with replacement [77]. Additionally, during the training of each tree, each split in the tree is generated from a random subset of the features. After training, test data predictions are generated by averaging the predictions from all the trees trained. These two extensions decrease the variance in the model to reduce the problem with overfitting in decision trees at the expense of slight increase in the bias [77].

In our implementation of distributed random forests, for each classification task, the h2o.ai function ‘randomForest’ was used with 200 trees, maximum depth of 20 levels and respective training and validation data for the task. The other parameters were set to their default values.

#### Gradient boosting (GB)

Gradient boosting machine is an ensemble method based on the idea that multiple weak learners can perform better than a single strong learner [78]. In the case of gradient boosting machines, the weak learners are short regression trees. At each iteration, a new tree is added that minimizes the loss function while keeping the other trees frozen. Additionally, at each iteration the loss function is modified such that training data points that were previously misclassified are weighted strongly. These methods are generally used as algorithms to rank a list such as in web search. This method is less susceptible to overfitting and does not suffer from the curse of dimensionality, however it is sensitive to noisy data and outliers [79].

In our implementation of gradient boosting, for each classification task, the h2o.ai function ‘gbm’ was used with 200 trees and maximum depth of 5 levels as per recommended range of 4 to 8 levels [80] wherein results were found insensitive to values chosen in this range.

#### Naïve Bayes (NB)

Naïve Bayes is a supervised classification method that constructs conditional probability distributions, p (C_k_ | X_1_,….,X_n_), for each category C_k_, where X_1_,….,X_n_ are the features used in training [81]. When new data is presented to the model, each category with the highest probability is assigned to the data point corresponding to unique features. It is a common baseline method for tasks such as text categorization, for identifying spams as well as in automatic medical diagnostics. This method makes a naïve assumption of independence of the features which may or may not be accurate given the nature of the data or process being modelled. However, these assumptions help it avoid the curse of dimensionality wherein an exponentially increasing amount of data is required with increasing features. In fact, the joint conditional probability distribution of the features can be calculated from the individual conditional feature distributions [81] .

In our implementation of Naïve Bayes, for each classification task, the h2o.ai function ‘naiveBayes’ was used with the default parameters and respective training and validation data for the task.

#### Variable importance

To examine which of the measured flow cytometry features were most important in the deep learning (feed forward artificial neural network with three hidden layers) analysis, the variable importance option was turned and visualized in h2o.ai’s Flow platform. Variable importance is notoriously difficult to compute for deep learning (feed forward artificial neural network with three hidden layers). The implemented method by Gedeon [82] considers the weights connecting the input features to the first two hidden layers. Our analysis was based on three flow cytometry features namely “FSC-A”, “SSC-A” and “AmCyan-A” corresponding to V1-V1000, V1001–2000 and V2001–3000 variables respectively in h2o Flow.

#### Receiver operating characteristic (ROC) curves

Area under the curve (AUC) analysis was performed for the clubbed data set on its test set split to test for robustness of the models. Since the models were multi class classification models, AUC values and receiver operating characteristic (ROC) curves were calculated by dividing the four-class prediction problem into four one-vs-all binary classification problems using the pROC package in R [83].

## Additional file

Additional file 1:  Experimental design, flow cytometry controls, hyperparameters and additional material. (DOCX 984 kb)

## Abbreviations

ACET: Acetate
ACETO: Putative Acetogens
ACIDO: Putative Acidogens
AUC: Area Under the Curve
BLAN: Blank sample (no carbon source)
BUTY: Butyrate
CELL: Cellulose
CNT: Carbon Nanotubes
COD: Chemical Oxygen Demand
CSV: Comma-Separated Values
DL: Deep Learning (feed forward artificial neural network with three hidden layers)
DRF: Distributed Random Forests
FCS: Flow Cytometry Standard
FENP: Ferrous Nanoparticles
FSC: Forward Scatter
GB: Gradient Boosting
GLUC: Glucose
HYDRO: Putative Hydrolyzers
MDS: Multidimensional Scaling
METHA: Putative Methanogens
MSE: Mean Square Error
NB: Naïve Bayes
NCH: Negative Control
NEWS: Newspaper Wastes
NGS: Next Generation Sequencing
NONE: No carbon source added
NP: Nanoparticles
PBS: Phosphate-buffered saline
PCA: Principal Component Analysis
PCH: Positive Control
PMT: Photomultiplying Tubes
PROP: Propionate
ROC: Receiver Operating Characteristic
SCOD: Soluble Chemical Oxygen Demand
SLUD: Sludge
SSC: Side Scatter
STVD: Starved (samples with no carbon source)
TC: Tetracycline
TCOD: Total Chemical Oxygen Demand
TINP: Titanium(IV) oxide Nanoparticles
TS: Total Solids
VFA: Volatile Fatty Acids
WFED: Well fed (all carbon sources combined)
