# Supplementary material for: Machine learning analysis of microbial flow cytometry data from nanoparticles, antibiotics and carbon sources perturbed anaerobic microbiomes
Source: J Biol Eng. 2018 Sep 12;12:19. doi: 10.1186/s13036-018-0112-9 (PMC6134764; doi:10.1186/s13036-018-0112-9)
Supplement: Supplementary file 1 — Experimental design, flow cytometry controls, hyperparameters and additional material. (DOCX 984 kb) [file 13036_2018_112_MOESM1_ESM.docx]

**Additional file 1**

**For**

**Machine Learning Analysis of Microbial Flow Cytometry Data from Nanoparticles, Antibiotics and Carbon Sources Perturbed Anaerobic Microbiomes**

Abhishek S. Dhoble^1^, Pratik Lahiri^2^, Kaustubh D. Bhalerao^*^

^1^Institutional Address: Department of Agricultural and Biological Engineering, University of Illinois at Urbana-Champaign, 1304 W. Pennsylvania, Urbana 61801, United States.

Email: dhoble2@illinois.edu

^2^Institutional Address: Department of Agricultural and Biological Engineering, University of Illinois at Urbana-Champaign, 1304 W. Pennsylvania, Urbana 61801, United States.

Email: plahiri2@illinois.edu

*Author for correspondence.

E-mail: bhalerao@illinois.edu, Tel: +1-217-244-6569; Fax: +1-217-244-0323

Address: 376 A Agricultural Engr Sciences Bld, 1304 W. Pennsylvania, Urbana Illinois 61801, United States.

Additional file 1

Table S1. Chemical properties of wastewater sludge.

| Total Chemical Oxygen Demand (TCOD) (mg/L) | 42,308 ± 3721 |
| --- | --- |
| Soluble Chemical Oxygen Demand (SCOD) (mg/L) | 5028 ± 865 |
| Total Solids (TS) (g/L) | 33.8 ± 0.9 |
| Volatile Solids (VS) (g/L) | 29.3 ± 1.1 |
| pH | 6.2 ± 0.7 |
| Ammonia (mg/L) | 180 ± 12.8 |

**Figure S1**

**
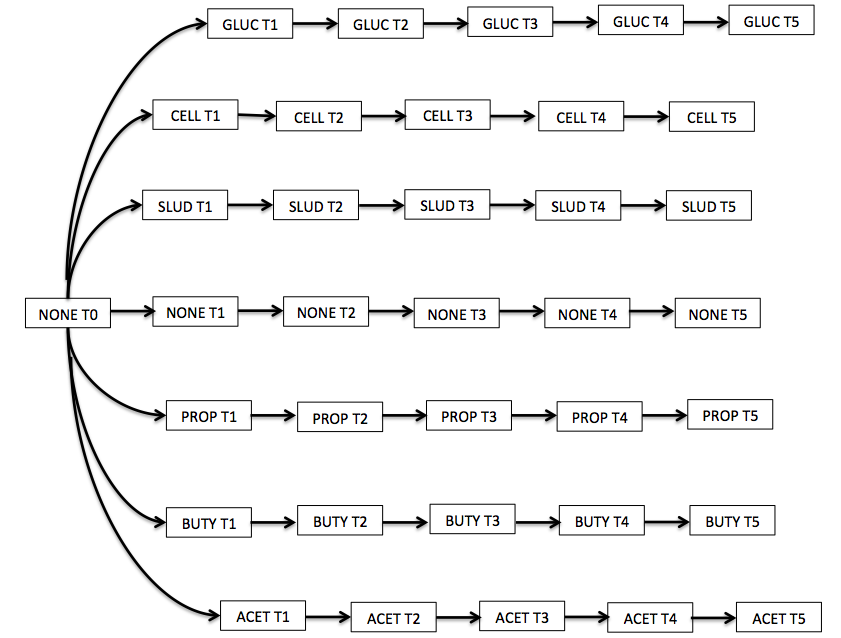
**

**Fig. S1.** Experimental design: glucose (GLUC), cellulose (CELL), propionate (PROP), butyrate (BUTY), acetate (ACET) , waste activated sludge (SLUD) and no carbon source (NONE) Number following ‘T’ represents the day.

**Figure S2**

**
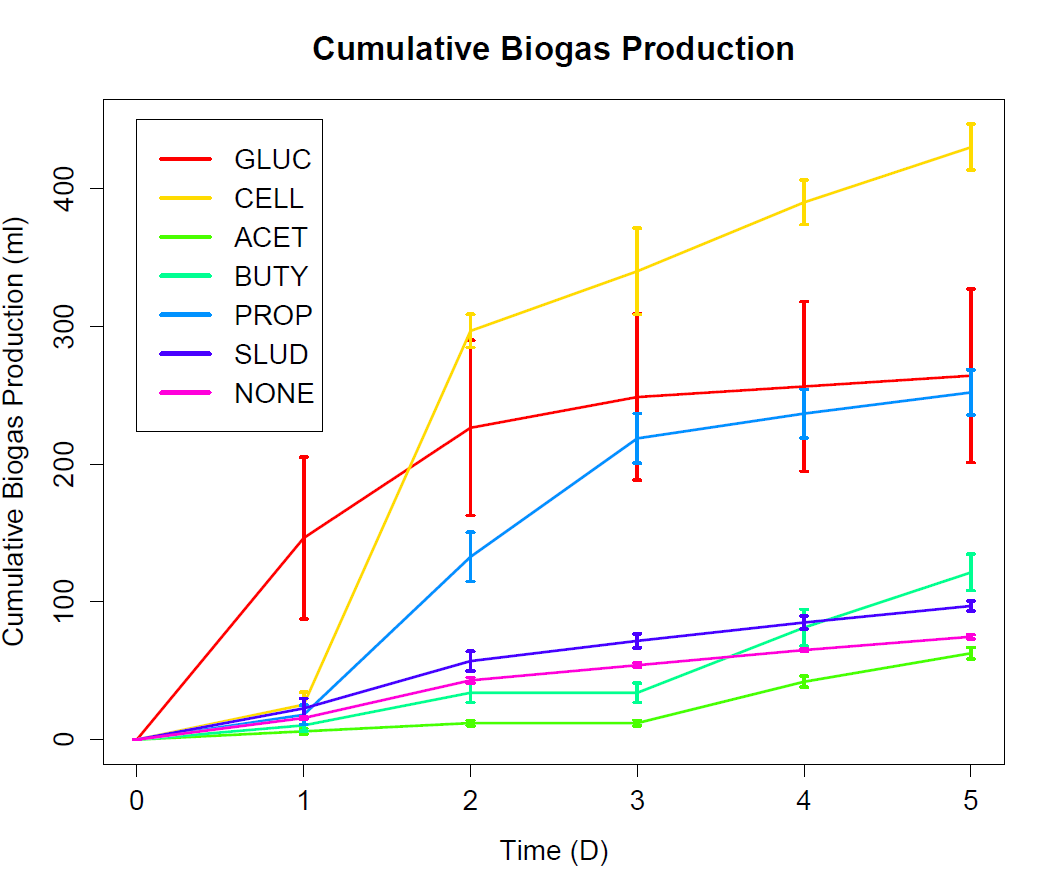
**

**
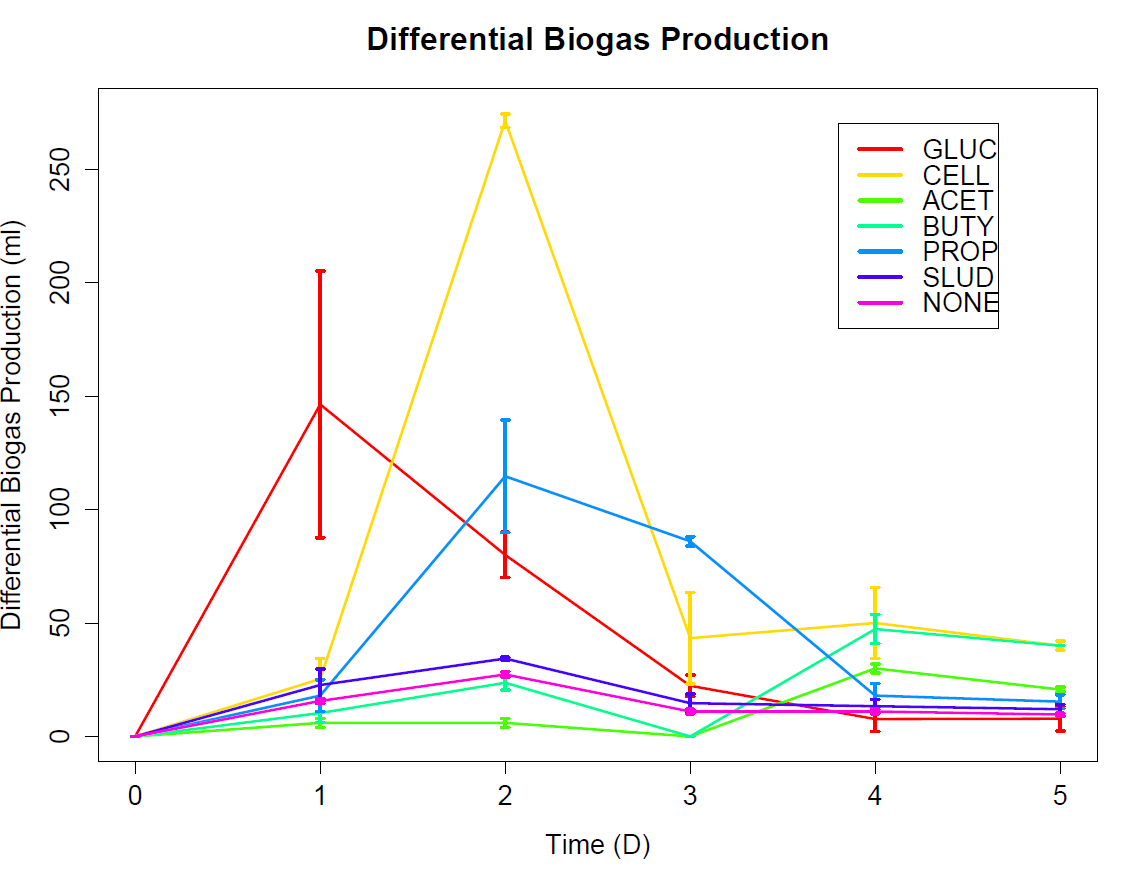
**

**Fig. S2.** Cumulative (top) and differential (bottom) biogas production from controlled addition of various carbon sources to mesophilic anaerobic systems.

**Figure S3**


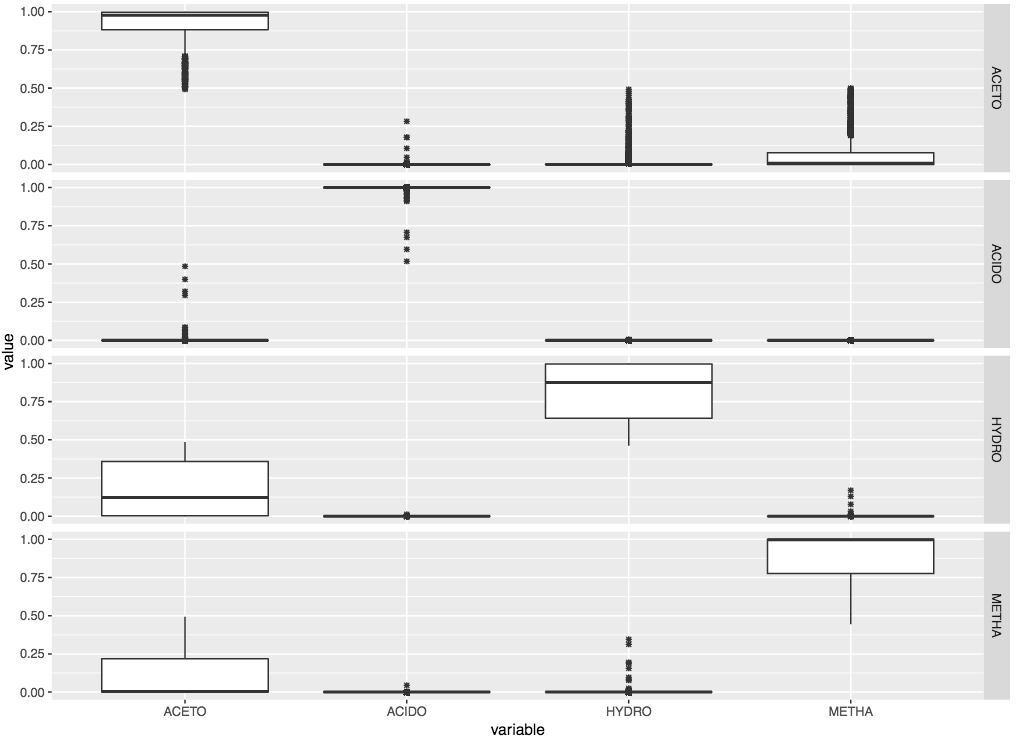


**Fig. S3.** Clubbed carbon sources predictions from H20.ai’s deep learning algorithm

**Figure S4**


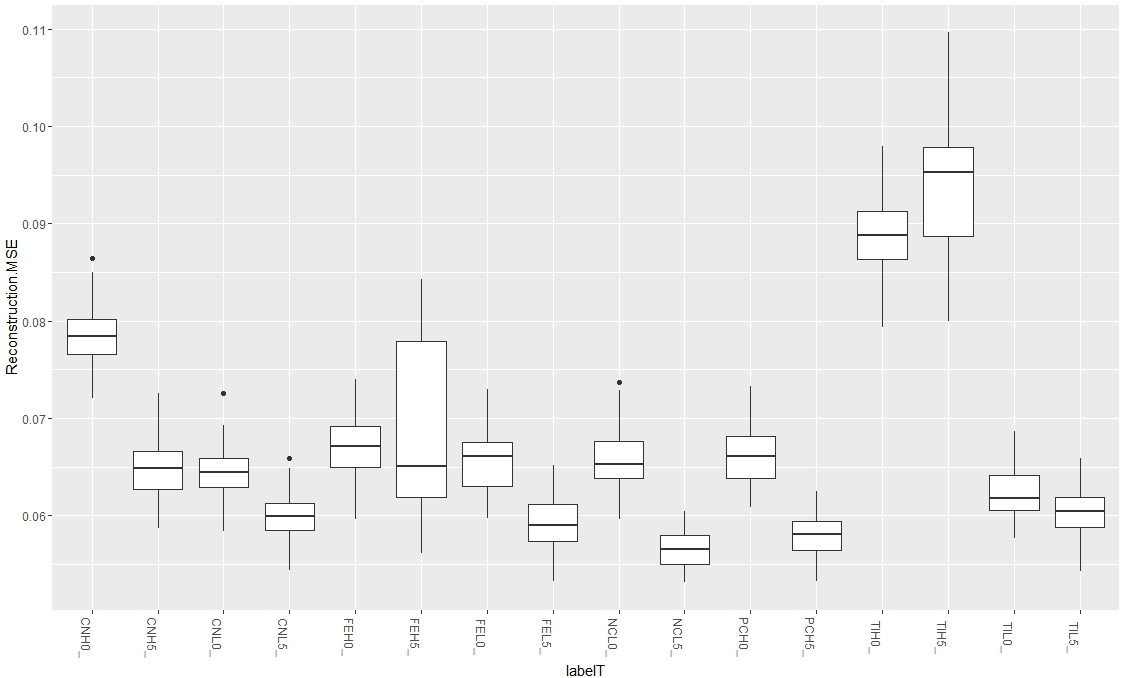


**Fig. S4.** Autoencoder MSE reconstruction for nanoparticles perturbed community

**Figure S5**


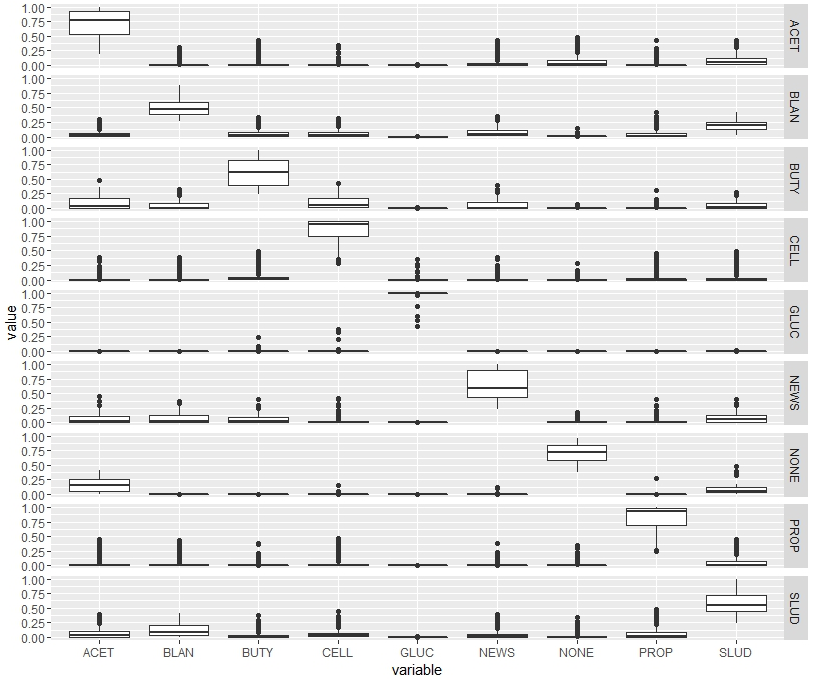


**Fig. S5.** Newsprint cytometric fingerprints predictions off carbon source data

## Figure S6 (a)


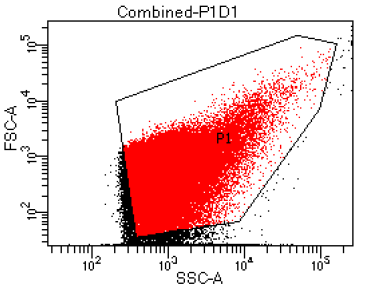


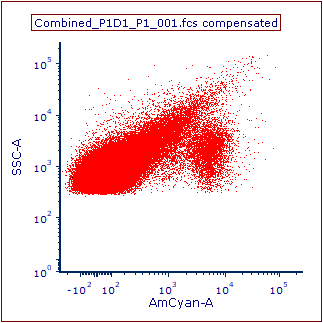

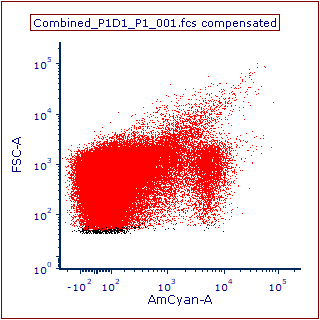


## Fig. S6. (a) The fixed gating template based on the control beads run used for exporting 100,000 events (shown in red P1) used in the analysis. Events in black representing background dead/junk cells along with high FSC events (putative doublets) were excluded from the analysis. An example of the gating including ‘AmCyan-A’ signals on the gated cells (red) has also been included.

## Fig. S6 (b)

##
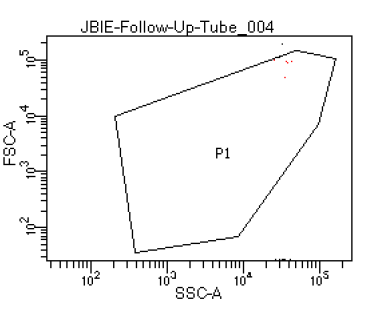

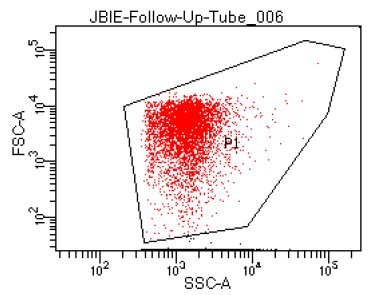

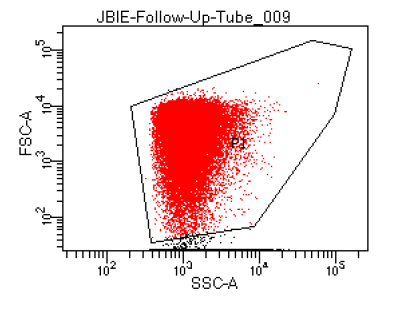


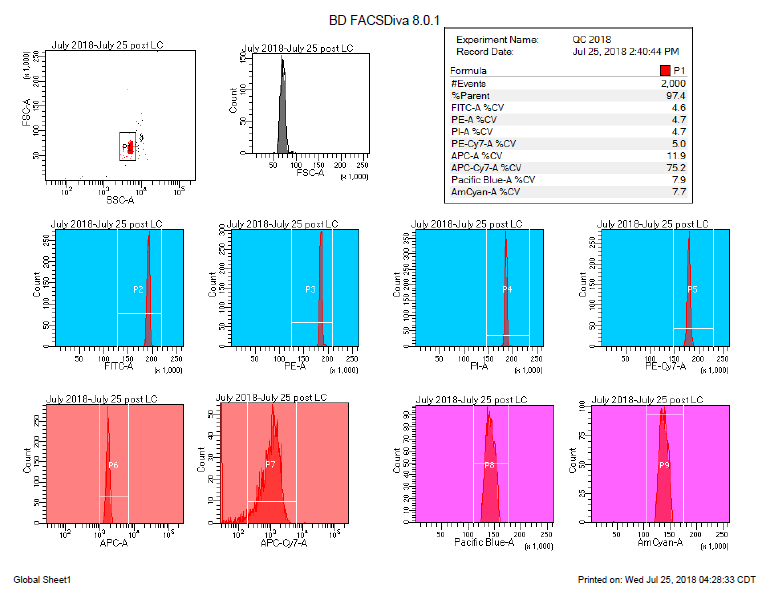


**Fig. S6. (b)** Demonstration of an incorporation of biologically relevant data with the fixed gating template. Tube 004 (PBS – A Negative control sample – only background data), Tube 006 (Quality Control - QC Beads from Flow-Check Flurospheres Lot#9434269F) and Tube 009 (*E. coli* HB 101) along with QC report for the beads used (shown in the bottom three rows). Lack of events (red dots) in Tube 004 and presence of them in Tubes 006 and 009 demonstrates the effectiveness of the demonstrated fixed gating template in capturing biologically relevant data.

## Fig. S6 (C)


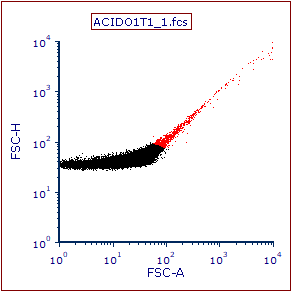

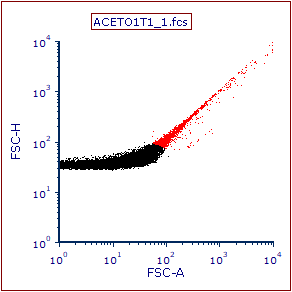


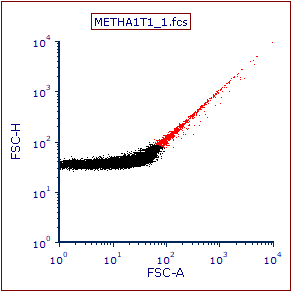

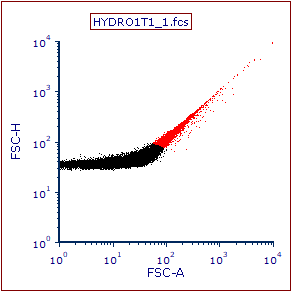


**Fig. S6. (c)** The area ('A') vs. height ('H') plots from the forward scatter (FSC) channel demonstrating diagonal orientation of the gated cells (red). The background data (black) has lower area values.

Table S2. Flow cytometry settings used in the experiments.

| Parameter | Voltage |
| --- | --- |
| FSC | 478 |
| SSC | 324 |
| AmCyan | 467 |

## Figure S7

##
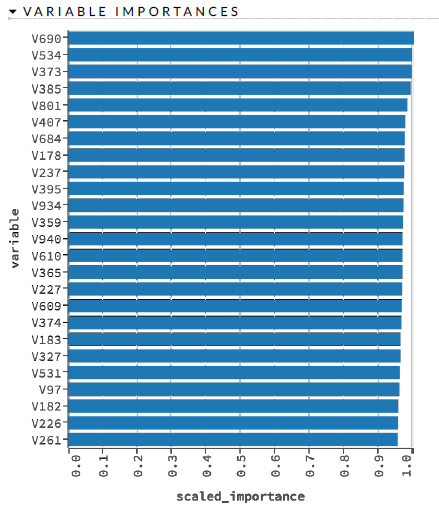


## Fig. S7. Variable importance output from H20.ai Flow depicting all the important variables are V<1000 which are forward scatter (FSC-A) variables. (V1001-2000: SSC-A; V2001-3000:AmCyan-A) for Deep Learning model on carbon sources dataset.

## Figure S8

##
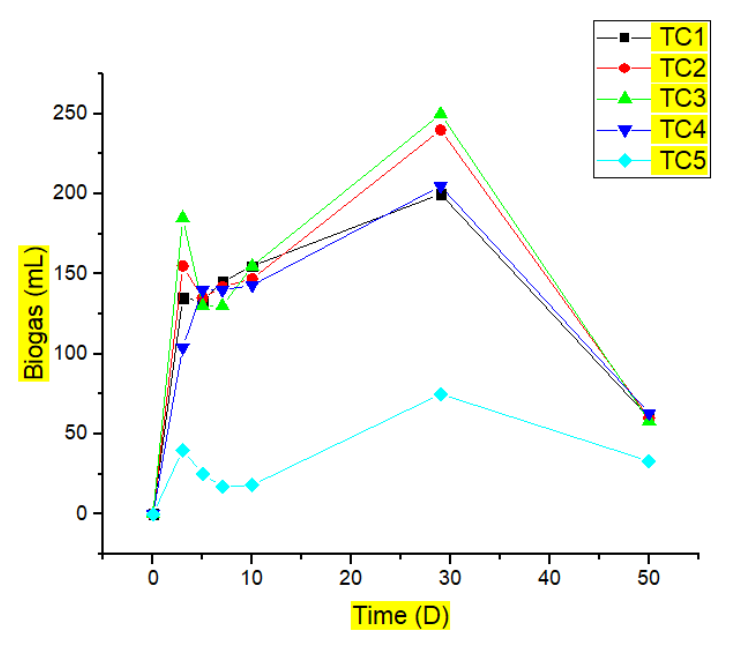


**Fig. S8.** Cumulative biogas production with samples perturbed with tetracycline at 20 mg/L (TC1), 40 mg/L (TC2) and 80 mg/L (TC3) concentrations along with positive (TC4) and negative (TC5) controls.

## Figure S9

**Fig. S9.** Multidimensional spacing (MDS) plots on flow cytometry samples from perturbation experiments with tetracycline at 20 mg/L (TC1), 40 mg/L (TC2) and 80 mg/L (TC3) concentrations along with positive (TC4) and negative (TC5) controls at different time points. As highlighted, samples on day 50 looks totally different than day 0.

Table S3. Confusion matrix of classification accuracies on the test set for a deep learning model trained on all the carbon sources fed individually (ACET, BUTY, CELL GLUC, PROP) and combined (WFED). STVD represents samples not fed with any carbon sources.

|  | ACET | BUTY | CELL | GLUC | PROP | STVD | WFED | Error |
| --- | --- | --- | --- | --- | --- | --- | --- | --- |
| ACET | 156 | 73 | 55 | 59 | 32 | 0 | 0 | 0.5840 |
| BUTY | 86 | 92 | 88 | 54 | 55 | 0 | 0 | 0.7547 |
| CELL | 68 | 86 | 120 | 37 | 61 | 1 | 2 | 0.6800 |
| GLUC | 86 | 38 | 31 | 207 | 13 | 0 | 0 | 0.4480 |
| PROP | 67 | 108 | 112 | 38 | 50 | 0 | 0 | 0.8667 |
| STVD | 0 | 0 | 0 | 0 | 0 | 130 | 245 | 0.6533 |
| WFED | 0 | 0 | 0 | 0 | 0 | 77 | 298 | 0.2053 |
| Total | 463 | 397 | 406 | 395 | 211 | 208 | 545 | 0.5989 |

**Fig. S10**


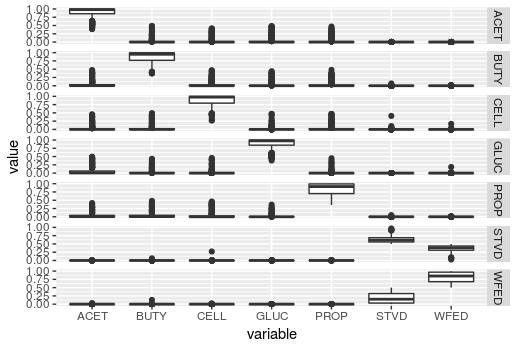


**Fig. S10**. Predictions on all the carbon sources fed individually (ACET, BUTY, CELL GLUC, PROP) and combined (WFED). STVD represents samples not fed with any carbon sources. WFED and STVD looks different than individual carbon ssources.

## A.1. Deep learning model run output for carbon sources

Model Details:

==============

H2OMultinomialModel: deeplearning

Model Key: model0

Status of Neuron Layers: predicting label, 8-class classification, multinomial distribution, CrossEntropy loss, 8,507,508 weights/biases, 97.9 MB, 50,635 training samples, mini-batch size 1

layer units type dropout l1 l2 mean_rate rate_rms momentum mean_weight weight_rms

1 1 3000 Input 10.00 %

2 2 2000 RectifierDropout 20.00 % 0.000010 0.000010 0.031988 0.010642 0.000000 0.001471 0.027578

3 3 1000 RectifierDropout 20.00 % 0.000010 0.000010 0.006230 0.009728 0.000000 -0.002646 0.026909

4 4 500 RectifierDropout 10.00 % 0.000010 0.000010 0.007733 0.061706 0.000000 -0.003057 0.036766

5 5 8 Softmax 0.000010 0.000010 0.022597 0.112614 0.000000 -0.007270 0.244480

mean_bias bias_rms

1

2 0.450454 0.026231

3 0.993666 0.004639

4 0.987295 0.006795

5 -0.201452 0.095198

H2OMultinomialMetrics: deeplearning

** Reported on training data. **

** Metrics reported on full training frame **

Training Set Metrics:

=====================

Extract training frame with `h2o.getFrame("h2otrain0")`

MSE: (Extract with `h2o.mse`) 0.0767435

RMSE: (Extract with `h2o.rmse`) 0.2770262

Logloss: (Extract with `h2o.logloss`) 0.3024106

Mean Per-Class Error: 0.09408333

Confusion Matrix: Extract with `h2o.confusionMatrix(<model>,train = TRUE)`)

=========================================================================

Confusion Matrix: Row labels: Actual class; Column labels: Predicted class

ACET BLAN BUTY CELL GLUC NONE PROP SLUD Error Rate

ACET 698 5 9 0 0 1 36 1 0.0693 = 52 / 750

BLAN 7 570 16 1 0 0 4 2 0.0500 = 30 / 600

BUTY 1 1 705 34 8 0 1 0 0.0600 = 45 / 750

CELL 0 1 9 740 0 0 0 0 0.0133 = 10 / 750

GLUC 0 0 0 0 750 0 0 0 0.0000 = 0 / 750

NONE 13 0 0 2 0 126 9 0 0.1600 = 24 / 150

PROP 0 0 0 0 0 0 500 0 0.0000 = 0 / 500

SLUD 154 18 72 26 0 0 30 450 0.4000 = 300 / 750

Totals 873 595 811 803 758 127 580 453 0.0922 = 461 / 5,000

Hit Ratio Table: Extract with `h2o.hit_ratio_table(<model>,train = TRUE)`

=======================================================================

Top-8 Hit Ratios:

k hit_ratio

1 1 0.907800

2 2 0.987400

3 3 0.996200

4 4 0.999200

5 5 0.999600

6 6 1.000000

7 7 1.000000

8 8 1.000000

H2OMultinomialMetrics: deeplearning

** Reported on validation data. **

** Metrics reported on full validation frame **

Validation Set Metrics:

=====================

Extract validation frame with `h2o.getFrame("h2ovalidate0")`

MSE: (Extract with `h2o.mse`) 0.4140956

RMSE: (Extract with `h2o.rmse`) 0.6435026

Logloss: (Extract with `h2o.logloss`) 2.155563

Mean Per-Class Error: 0.481

Confusion Matrix: Extract with `h2o.confusionMatrix(<model>,valid = TRUE)`)

=========================================================================

Confusion Matrix: Row labels: Actual class; Column labels: Predicted class

ACET BLAN BUTY CELL GLUC NONE PROP SLUD Error Rate

ACET 175 28 84 6 0 2 66 14 0.5333 = 200 / 375

BLAN 47 84 72 18 0 0 61 18 0.7200 = 216 / 300

BUTY 36 40 182 95 8 0 13 1 0.5147 = 193 / 375

CELL 13 14 42 275 0 0 24 7 0.2667 = 100 / 375

GLUC 0 0 0 0 375 0 0 0 0.0000 = 0 / 375

NONE 30 0 0 8 0 20 17 0 0.7333 = 55 / 75

PROP 9 7 7 9 0 0 214 4 0.1440 = 36 / 250

SLUD 115 62 79 32 0 0 63 24 0.9360 = 351 / 375

Totals 425 235 466 443 383 22 458 68 0.4604 = 1,151 / 2,500

Hit Ratio Table: Extract with `h2o.hit_ratio_table(<model>,valid = TRUE)`

=======================================================================

Top-8 Hit Ratios:

k hit_ratio

1 1 0.539600

2 2 0.749200

3 3 0.854800

4 4 0.935600

5 5 0.978400

6 6 0.998400

7 7 0.999200

8 8 1.000000

Scoring History:

timestamp duration training_speed epochs iterations samples training_rmse

1 2018-02-06 15:35:06 0.000 sec 0.00000 0 0.000000

2 2018-02-06 15:35:34 40.500 sec 89 obs/sec 0.48160 1 2408.000000 0.78138

3 2018-02-06 15:37:47 2 min 53.296 sec 97 obs/sec 2.88800 6 14440.000000 0.53954

4 2018-02-06 15:39:53 4 min 59.707 sec 100 obs/sec 5.30420 11 26521.000000 0.27703

5 2018-02-06 15:41:57 7 min 2.959 sec 102 obs/sec 7.71060 16 38553.000000 0.32203

6 2018-02-06 15:44:02 9 min 7.815 sec 103 obs/sec 10.12700 21 50635.000000 0.15640

7 2018-02-06 15:44:14 9 min 19.952 sec 103 obs/sec 10.12700 21 50635.000000 0.27703

training_logloss training_classification_error validation_rmse validation_logloss

1

2 3.43458 0.65220 0.79049 3.64349

3 1.22559 0.33480 0.69416 2.37369

4 0.30241 0.09220 0.64350 2.15556

5 0.47280 0.12460 0.68741 3.21005

6 0.10803 0.02980 0.65320 3.03252

7 0.30241 0.09220 0.64350 2.15556

validation_classification_error

1

2 0.66000

3 0.53440

4 0.46040

5 0.51080

6 0.46160

7 0.46040

## A.2. Cross Validation (CV) results

Hyperparameters for Nested CV:

**Gradient Boosting (GB):**

- Shrinking the contribution of each tree by learning rate: ‘learning_rate’: [0.001, 0.01, 0.1, 0.15],
- Number of features to consider when looking for best split: 'max_features': ['sqrt', 'auto'],
- Minimum number of samples required to be the leaf node: 'min_samples_leaf': [50, 75, 100, 200, 500, 100],
- Minimum number of samples required to split an internal node: 'min_samples_split': [2, 5, 10, 50, 100, 200, 500, 1000],
- Number of boosting stages to perform: 'n_estimators': [50, 100, 500, 1000, 2000]

**Distributed Random Forests (DRF):**

- Number of features to consider when looking for best split: 'max_features': ['sqrt', 'auto'],
- Minimum number of samples required to be the leaf node:'min_samples_leaf': [50, 75, 100, 200, 500, 100],
- Minimum number of samples required to split an internal node: 'min_samples_split': [2, 5, 10, 50, 100, 200, 500, 1000],
- Number of trees in the forest: 'n_estimators': [50, 100, 500, 1000, 2000]

**Naïve Bayes (NB):**

- NB has no hyperparameters to tune.

**Deep Learning* (DL) (*(feed forward artificial neural network with three hidden layers):**

- Number of passes over the entire training dataset: 'epochs': [50, 100, 150],
- Fraction of nodes ignored in each layer: 'dropout': [[0.1, 0.1, 0.1], [0.2, 0.2, 0.2], [0.4, 0.4, 0.4]]

Table S4. Cross validated (5-fold) model scores on classifying putative functional groups (without tuning hyperparameters; accuracy values on the test set with tuned hyperparameters are presented in Table 1).

| Method | Accuracy (%) | Standard Deviation |
| --- | --- | --- |
| Deep Learning (DL) | 64.34 | 3.7 |
| Distributed Random Forests (DRF) | 59.85 | 0.45 |
| Gradient Boosting (GB) | 67.19 | 0.28 |
| Naive Bayes (NB) | 61.24 | 0.3 |

**A.3. Population vs Sample Distribution Characteristics**

**Fig. S10**


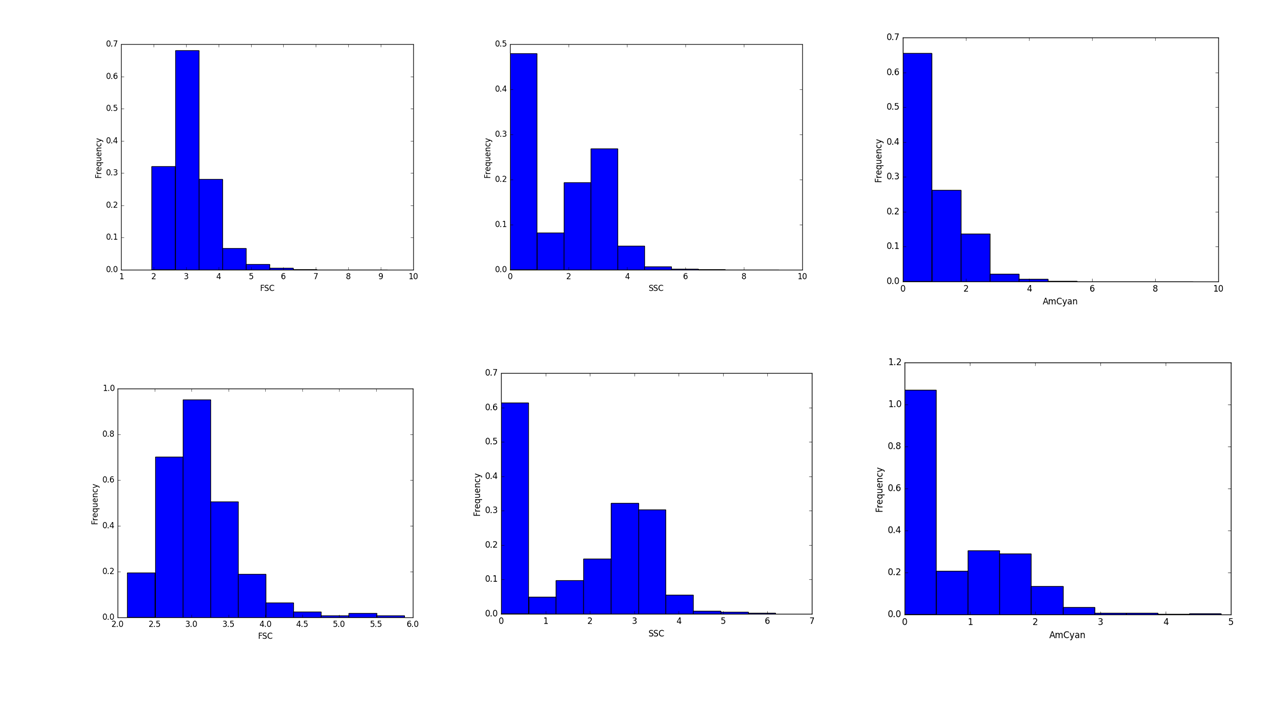


**Fig. S10.** The distribution of “FSC-A", "SSC-A" and "AmCyan-A” values (L to R) for all methanogens groups in the test set (top row) as well as the distribution of the same values in one randomly chosen vector of 1000 events (bottom row).
